# Increasing Participation Rates in Germany’s Skin Cancer Screening Program (HELIOS): Protocol for a Mixed Methods Study

**DOI:** 10.2196/31860

**Published:** 2021-12-13

**Authors:** Theresa Steeb, Markus V Heppt, Michael Erdmann, Anja Wessely, Stefanie J Klug, Carola Berking

**Affiliations:** 1 Department of Dermatology University Hospital Erlangen Friedrich-Alexander-University Erlangen-Nuremberg Erlangen Germany; 2 Comprehensive Cancer Center Erlangen – European Metropolitan Region of Nuremberg Erlangen Germany; 3 Chair of Epidemiology Department of Sport and Health Sciences Technical University of Munich Munich Germany

**Keywords:** skin cancer, melanoma, squamous cell carcinoma, basal cell carcinoma, screening, early detection, focus group, mixed methods, cross-sectional study, prevention

## Abstract

**Background:**

In 2008, a nationwide skin cancer screening (SCS) program was implemented in Germany. However, participation rates remain low.

**Objective:**

The overall objective of the HELIOS study is to identify subgroup-specific invitation and communication strategies to increase informed SCS participation in Germany.

**Methods:**

Focus group discussions will be performed in Erlangen, Germany, to explore potential invitation and communication strategies as well as possible barriers and motivating factors to participate in SCS. Male and female patients of different age groups who have already been diagnosed with skin cancer, as well as participants without a prior diagnosis of skin cancer, will be invited. Based on these results, an online questionnaire will be developed to identify subgroup-specific invitation strategies. A random sample of 2500 persons from the general population aged >35 years from the Munich area will be contacted to complete the questionnaire. Besides descriptive analysis, multinomial logistic regression will be performed. Additionally, a cluster analysis will be conducted to discover patterns or similarities among the participants.

**Results:**

Recruitment for the focus group studies started in February 2021 and is ongoing. As of August 2021, we have enrolled 39 participants. We expect to end enrollment in the qualitative study in September 2021 and to finish the analysis in December 2021. The second part of the study will then start in January 2022.

**Conclusions:**

The results of this project will enable us to derive improved and more efficient invitation and communication strategies for SCS. These may be implemented in the future to facilitate increased SCS uptake and early skin cancer detection.

**International Registered Report Identifier (IRRID):**

DERR1-10.2196/31860

## Introduction

Skin cancer is one of the most frequently diagnosed cancer entities in Germany. The incidence of melanoma and nonmelanoma skin cancer has steadily increased in recent years [1]. Besides reducing exposure to ultraviolet radiation by means of sun protection measures, early detection of suspicious skin lesions represents a key secondary prevention strategy [2,3]. Aiming to reduce skin cancer–associated mortality and morbidity, a national skin cancer screening (SCS) program was introduced in Germany in July 2008. It involves a voluntary, standardized full-body examination by dermatologists or general practitioners who have been specifically trained for this purpose. As a part of this examination, risk factors for skin cancer as well as prevention measures are addressed. The costs are reimbursed by all German statutory health insurance companies biannually for members who are >35 years, while some health insurance companies also cover SCS costs for younger members [4]. The decision to implement SCS at the population level was based on the results of the pilot SCREEN (Skin Cancer Research to Provide Evidence for Effectiveness in Northern Germany) project [5]. Following this study, there was both a significant decrease in melanoma mortality [6] and a shift in the T-stage distribution in favor of thin melanomas [7]. These differences in comparison to neighboring regions without SCS suggested that they were attributable to SCS, but this has been subject to debate [6,8].

Since its introduction, more than 13 million patients have participated in the SCS program, and estimated participation rates have ranged between 24% and 39% [4,9-11]. However, this highlights that about 60% to 75% of eligible residents in Germany have never taken advantage of the SCS program. Nevertheless, most people appreciate the option to participate in the SCS program and, additionally, informed persons use SCS more frequently than uninformed persons [9]. Moreover, women undergo SCS more frequently than men [12]. However, unlike the organized invitation programs for mammography, cervical cancer, and colorectal cancer screening, there are currently no campaigns or target group–specific invitation strategies aimed at increasing people’s decision to participate in SCS in Germany.

In order to develop target group–specific invitation strategies to ultimately increase the SCS participation rate in Germany, we initiated the HELIOS study (German acronym for *Hautkrebsspezifische Einladungsverfahren zur informierten Screeningteilnahme*, or in English, Skin Cancer–Specific Invitation Strategies to Participate in the Skin Cancer Screening Program). Here, we describe the study design and summarize the study protocol. The results of our project will contribute to increase participation rates in SCS and thus lead to earlier detection of skin cancer.

## Methods

### HELIOS Project

**Figure 1:**
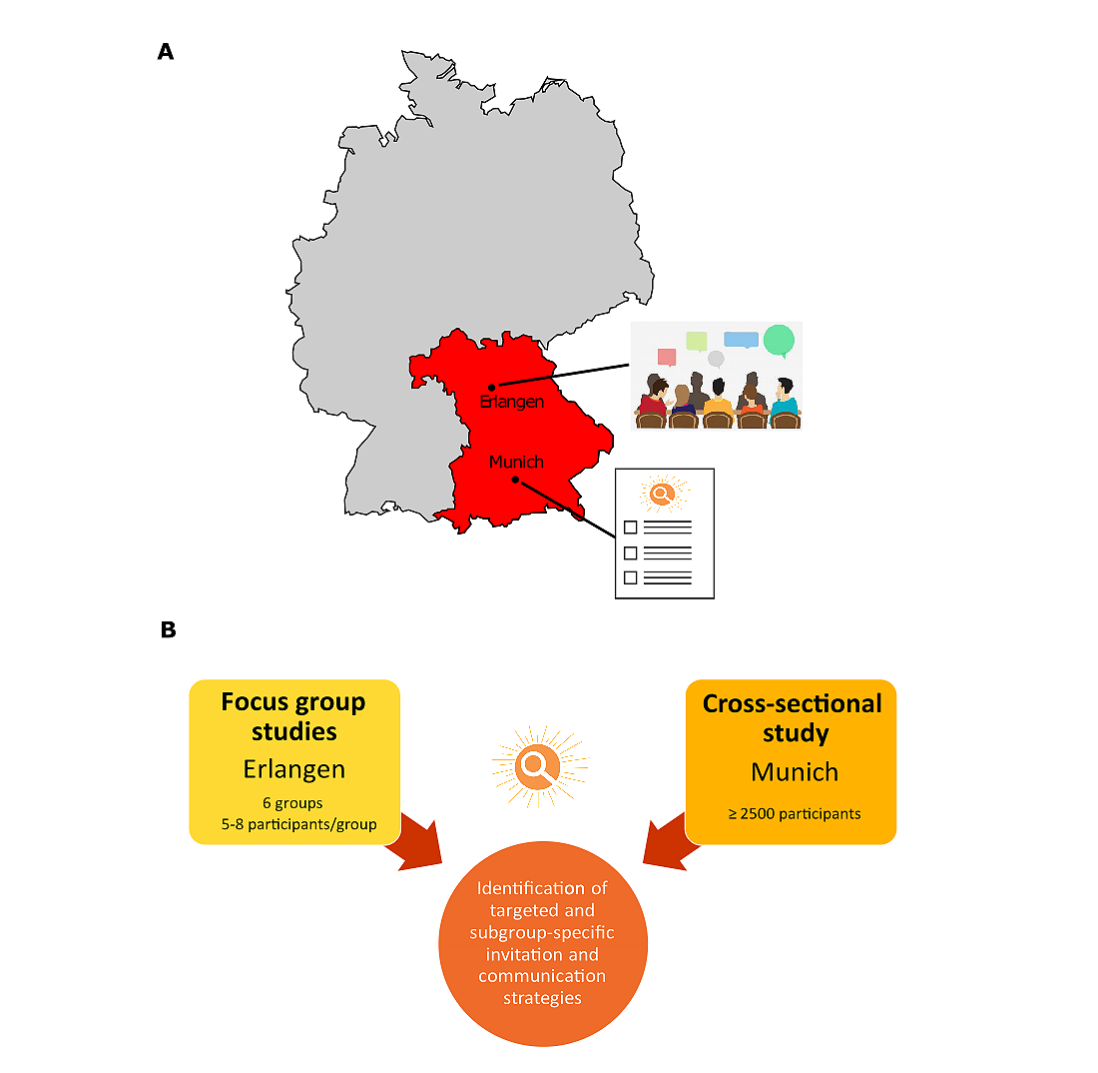
(A) Map of Germany showing the location of the 2 subprojects within the federal state of Bavaria. (B) Overview of the study flowchart of the subprojects within the HELIOS project.

The HELIOS project comprises two discrete, yet complementary, subprojects. The first part consists of a qualitative approach comprising several focus group sessions with different subgroups in Erlangen, Germany, to exploratively collect possible communication strategies for SCS. The second part is based on the results of the focus group sessions and involves a cross-sectional study among a random sample of residents in Munich, Germany, aimed at identifying suitable, targeted communication strategies for different subgroups (Figure 1).

### Part 1: Focus Group Study

#### Objective and Study Design

In order to exploratively collect possible invitation procedures and communication approaches for different subgroups, a qualitative and explorative design will be used through focus group discussions. As a part of these discussions, the already existing information brochure *Detecting Skin Cancer*, of the German Cancer Aid, will be additionally evaluated in order to obtain target group–specific suggestions for improvement [13].

Qualitative approaches facilitate an in-depth picture of patients’ preferences and needs [14,15]. In addition, the interactive component of the focus groups enables participants to ponder, reflect, and listen to the experiences and opinions of others [16]. The interview will be structured according to published guidelines for focus groups [16]. A manual with questions for the focus groups will be developed by the investigators of this study and will be based on an assessment of the literature and dermato-oncologic experience.

The focus group interviews will assess the following aspects:

1. Willingness to participate in the SCS program with reasons for or against participation;
2. Interest in invitation procedures and preferred communication strategy;
3. Information required for an informed decision for or against participation in the SCS program;
4. Evaluation of the German Cancer Aid’s information brochure Detecting Skin Cancer [13] to obtain patient-specific suggestions for improvement.

Follow-up and probing questions will be used for clarification and elaboration. This subproject is particularly important as it takes into account the perspective and view of both participants who have not been diagnosed with skin cancer and patients with skin cancer. The semistructured interview guide is available in Multimedia Appendix 1.

#### Sampling, Recruitment, and Data Collection

Participants will be recruited via German patient support groups (eg, Hautkrebs-Netzwerk Deutschland, Melanom Info Deutschland), local Facebook groups, as well as through in-hospital flyers and direct contact by attending physicians in the Oncological Outpatient Department of the university hospital (Friedrich-Alexander-Universität Erlangen-Nürnberg). Patients who have already been diagnosed with skin cancer, as well as healthy or previously unaffected participants, will be eligible to participate in the discussions. A total of 6 focus groups of 5 to 8 participants, each with a duration of 45 to 90 minutes, are planned (a total of 30 patient or participant representatives) in Erlangen. The respective groups will differ in terms of gender composition (male/female) and age (35-50 years, 51-65 years, and >65 years). The participants will receive a financial incentive for successful participation. The interviews will be audiorecorded and moderated by an experienced interviewer and assistant. Demographic data such as age or gender will be obtained in advance from the participants using an anonymized questionnaire.

#### Data Analysis

All sessions will be transcribed verbatim and analyzed by 2 investigators independently, presumably via a qualitative content analysis according to Mayring [16] with the aid of the software MAXQDA (VERBI Software). The transcribed data will not be linked to any patient-identifying information to assure anonymity. Sociodemographic data will be presented descriptively as frequencies (%); age will be presented as mean or median and range. Prior to the focus groups, we will collect informed consent from each participant. We will closely adhere to the Consolidated Criteria for Reporting Qualitative Research (COREQ) checklist [17].

### Part 2: Cross-sectional Study

#### Objective and Study Design

The aim of the second subproject is the identification of target group–specific invitation procedures for the SCS program by means of an anonymized, questionnaire-based cross-sectional study in which the previously identified relevant invitation procedures from the focus group discussions (eg, postal invitation, invitation via email) will be further investigated. The invitation procedures will be correlated with the respective sociodemographic profile of the participants to create a prediction model from which the preferred invitation procedure can be derived. The following questions will therefore be answered:

1. Which subgroups prefer which invitation procedure?
2. Have different personal factors such as age, gender, social status, or family background influence or impact the preferred invitation procedure and willingness to participate in the SCS program?

#### Inclusion Criteria

Adult residents with a primary residence in the city of Munich aged >35 years will be included. Furthermore, participants need to have sufficient German-language skills to understand the general information provided about the project and to complete the online questionnaire.

#### Survey Dissemination

A random sample of the general population aged >35 years from the Munich area will be drawn from the local population registration office. The selected population will receive invitation letters with personalized 6-digit passwords (consisting of letters and numbers), which allow access to the online questionnaire. Additionally, the invitation letter will include a scannable QR code directly leading to the online questionnaire. Before answering the questionnaire, participants will have to confirm that they have read the general information provided online, including information about data protection. After this step, informed consent will be obtained online. Those who do not respond to the invitation will not be contacted again. The recruitment period is estimated to take 4 months. The questionnaire will be made available to the participants through a web-based survey tool, such as SurveyMonkey (Momentive Inc) or LimeSurvey (Carsten Schmitz and LimeSurvey Team). Each participant contacted may only take part in the survey once. The questionnaires will be numbered consecutively for data entry but will not be linked to participant-identifying information to ensure irreversible anonymity. We chose Munich as it is the capital city of Bavaria and has a larger number of available residents. In addition, we believe that the project will benefit from an intercity design as views from a medium-sized city (Erlangen) and one of the largest cities in Germany (Munich) will be included. No incentive will be offered for completion of the survey.

#### Questionnaire Development

Since there are currently no validated survey instruments tailored to the aim of our study, the questionnaire will be developed de novo based on the results from the focus group discussions as well as an assessment of the literature and dermato-oncological expertise. The questionnaire will consist of a multiple-choice format and will address sociodemographic data as well as previous participation in the SCS program. Further questions will elaborate the participants’ preferred invitation and communication procedure. Before final dissemination, the questionnaire will be pretested and validated for clarity and comprehension by independent researchers who will not be involved in the design of the original questionnaire and volunteering patients from University Hospital Erlangen. Unclear items will be thoroughly discussed and rephrased until a consensus on clarity is reached. Based on this feedback, the questionnaire will be revised to its final version. The first draft of the questionnaire can be obtained from Multimedia Appendix 2.

#### Data Analysis and Sample Size Calculation

The sample size calculation is based on a significance level of α=.05 with a minimum effect size of 30% and a response rate of 20% in Munich. At least 500 questionnaires are needed to detect an actual significant effect; therefore, at least 2500 participants should be contacted since we expect a low response rate of 20%. Descriptive analysis and multinomial logistic regression models will be performed to identify relevant correlations. The preferred invitation procedure will be used as the dependent variable, while age, willingness to participate, and other risk factors will serve as independent variables. Furthermore, chi-square tests or exact Fisher tests will be performed to investigate correlations between the sociodemographic variables and the individual questions. To counteract the problem of multiple testing, the Bonferroni correction method will be applied. A cluster analysis will be performed to discover patterns or similarities within the participants. Categorical variables will be expressed as frequencies and percentages, and continuous variables as median and range. A *P* value <.05 will be considered as statistically significant. The statistical analyses will be performed with SAS (SAS Institute). This subproject will be guided by the Strengthening the Reporting of Observational Studies in Epidemiology (STROBE) statement [18].

## Results

The study was approved by the institutional review board of University Hospital Erlangen (August 2020). The manual for the focus group discussions has been created. Recruitment for the focus group sessions started in February 2021 and is ongoing. We expect to end enrollment in the qualitative study in September 2021 and to finish the analysis in December 2021. The second part of the study will then start in January 2022. This study is expected to conclude in the summer of 2022.

## Discussion

The HELIOS project aims to determine target group–specific invitation strategies to ultimately facilitate an increase in SCS participation rates in Germany, and thus lead to an earlier detection of skin cancer. Based on the results, suitable and motivating invitation and communication procedures can be implemented in the future. Therefore, our study will contribute to the realization of the goals of the National Cancer Plan in the field of dermato-oncology. In addition, the HELIOS project should contribute to the realization of the goals of the SCS such as the reduction of mortality and morbidity in Germany. Importantly, this project consists of 2 distinct subprojects in order to include the views of both patients with skin cancer and unaffected individuals.

While SCS was implemented more than 10 years ago based on the evidence derived from the results of the SCREEN study, SCS acceptance at the population level remains highly controversial [19,20]. SCS itself is noninvasive and does not cause any relevant damage. However, unnecessary biopsies as a result of false-positive examinations and overdiagnosis need to be kept in mind. A further point of criticism is that no reduction in melanoma mortality has been observed thus far after the implementation of the nationwide SCS, which contrasts with the results of the pilot project [21]. The reasons for this discrepancy remain unclear. One possible reason is that the performance of the 2 screening programs differ in some aspects [22]. Whereas in the national SCS only dermatologists and general practitioners conduct the screening, in the SCREEN project general practitioners, gynecologists, urologists, and surgeons were also involved. Furthermore, the initiators of the SCREEN study concluded that the preceding public promotion prior to the implementation of the SCREEN project may have contributed to the success of the project [6].

In Germany, mammography screening is currently performed as an organized screening program. It was established between 2005 and 2009 and is based on an invitation system. All women between the ages of 50 and 69 years are invited to get screened with a mammography every 2 years and are informed about the screening offer by means of a leaflet. The overall participation rate among 5,528,937 invited women in 2015 was 51.5%. Depending on the federal state, the participation rates ranged between 43% and 63% [23]. In a cross-sectional study with 13,144 women in 2014-2015, an even higher participation rate with 74.2% was described. Of these, 80.7% cited the invitation letter to account for participation [24]. Since July 2019, colorectal cancer screening has been implemented as a nationwide, risk group–adapted, organized program in Germany [25]. Following the promising participation rates of mammography in Germany as well as the decision to implement colorectal cancer screening and cervical cancer screening as an organized screening program, it is necessary to also investigate and derive communication strategies for SCS to increase participation rates.

A potential barrier to carrying out the focus group discussion in the first subproject is participant recruitment. If an insufficient number of people agree to participate, the recruitment period will have to be extended. In a next step, we will have to adjust the subgroup distribution and broaden, for example, the age restrictions. If participant numbers still remain low, we will have to critically discuss within our project coordination team whether we will perform focus group discussions with less than 5 persons or whether we will perform individual interviews. Due to the current COVID-19 pandemic and the subsequent social-distancing regulations, we are unsure whether focus group discussions are feasible. Nevertheless, we are confident that we can assemble a desirable number of participants for the discussions since we will use various recruitment strategies, such as patient support groups (including a Facebook group with more than 2000 active members), active contact through physicians, and flyers. Besides this, participants will receive an incentive, which will also be a motivation to engage in the discussions. Additionally, recruitment for the second subproject bears the risk of a low response rate since residents in the Munich area are often skeptical of surveys [26,27]. To counteract a small sample size and the consequent underpowering of our results, we will contact at least 2500 participants in order to obtain at least 500 data sets. Further, we will provide a scannable QR code that directly leads to the online questionnaire in order to facilitate accessibility.

Overall, the results of the HELIOS study will enable us to derive suitable evidence-based invitation and communication strategies for SCS. These may be implemented in the future to facilitate increased SCS uptake and early skin cancer detection.

## Appendix

Multimedia Appendix 1 Focus group guide for part 1 of the project.

Multimedia Appendix 2 Draft of the questionnaire for part 2 of the project.

## Abbreviations

COREQ: Consolidated Criteria for Reporting Qualitative Research
HELIOS: Hautkrebsspezifische Einladungsverfahren zur informierten Screeningteilnahme (Skin Cancer–Specific Invitation Strategies to Participate in the Skin Cancer Screening Program)
SCREEN: Skin Cancer Research to Provide Evidence for Effectiveness in Northern Germany
SCS: skin cancer screening
STROBE: Strengthening the Reporting of Observational Studies in Epidemiology
