# Supplementary material for: Increasing Participation Rates in Germany’s Skin Cancer Screening Program (HELIOS): Protocol for a Mixed Methods Study
Source: JMIR Res Protoc. 2021 Dec 13;10(12):e31860. doi: 10.2196/31860 (PMC8713106; doi:10.2196/31860)
Supplement: Multimedia Appendix 1 [file resprot_v10i12e31860_app1.docx]

**Semi-structured guide for the focus group studies (HELIOS) Version 1.0**

- - Welcome
- - Introduction
- - Explanation of the research project
- - Request for consent

| **Guiding question, narrative impulse** | **Memo** | **Questions to keep the conversation flowing** |
| --- | --- | --- |
| **TOPIC 1: Willingness to participate in the HKS with reasons for/against participation.**  **Aim: Evaluation of the participants' attitude towards the HKS.** | | |
| **Interviewer: Short introduction to skin cancer screening**  **Please tell me if you have ever heard of skin cancer screening and what do you think about it?**  **Here, if necessary, brief clarification about SCS, if no one should have heard of it so far.** | - Have you ever participated in skin cancer screening?   o If yes, why?  o If no, why not?   - Where have you participated in skin cancer screening? Has a dermatologist or primary care physician ever examined all of your skin for abnormal moles? Preference for dermatologist or primary care physician?/preferences? - How often do you participate in skin cancer screening? - How did you feel or how would you feel at SCS? Did you find anything particularly unpleasant or positive? Why was the unpleasant/positive? - What are your expectations of SCS? - What fears or concerns do you associate with SCS? | - Can you describe that in more detail? - How exactly did it come about? - I would still be interested to know if... - What was that like for you/how did you feel about it? - Can you describe that to me in a little more detail? - What was the next step? - Why? - Can I ask again if I understood this correctly from you...? - Did I understand you correctly that...? - So you understand it like this... |
| **TOPIC 2: Interest in invitation procedures and preferred form of invitation.**  **Goal: Identification/collection of potential invitation or communication procedures.** | | |
| **How did you learn about the possibility of SCS?**  **Or rather:**  **How would you like to hear about the possibility of SCS?** | - Did you learn about SCS from doctor/friends/acquaintances/health insurance/newspaper/internet? - Have you looked up information about SCS from sources other than your doctor/physician? If so, please let me know where you looked and what was helpful to you? - Do you have any ideas on how (through what channels) to reach many people to learn about SCS? - What is your opinion on an invitation procedure with letter and fixed appointment, similar to mammography screening? ( show letter/invitation procedure) - What is your opinion of a reminder via phone call? - What is your opinion on a reminder via SMS? - What is your opinion of a reminder via email? - Would you be willing to download a reminder app on your smartphone? | - What happened next? - What do you associate with it? - Why? - I would still be interested to know if... - Can you describe that to me in a little more detail? - Can you go into more detail? - Can you give an example of this? - Can I ask again if I understood this correctly from you...? - Did I understand you correctly that...? - You also understand this in the same way... |
| **TOPIC 3: Required information**  **Goal: Identify information needed (important vs. unimportant) to make an informed decision.** | | |
| **What information would you like to have in order to decide whether or not to participate in SCS?**   - **Which information was/would be helpful for you? And which information was/would not be helpful for you?** | - Was there any information you might have liked to know about SCS opportunities for which you could not get answers or that was not available? - In what ways were you told or provided information about SCS opportunities that were helpful to yourself? - Is there anything that helped or did not help you in obtaining or understanding information about SCS options that I did not ask about? If so, could you describe this for me? - Do you have any ideas about how to motivate more people to participate in SCS? - Is there anything that doctors, policy makers, or anyone else could do about it? - What other professional or population groups do you think could be involved? - What are your wishes for the SCS for the future? | - Can you describe this in more detail? - I would still be interested in whether... - What was that like for you/how did it make you feel? - Can you describe that to me in a little more detail? - What was the next step? - Why? - Can I ask again if I understood this correctly from you...? - Did I understand you correctly that...? - So you understand it like this... |
| **TOPIC 4: Evaluation of the chapter on SCS in the early detection leaflet and possible suggestions for improvement.**  **Goal: Derive positive or negative aspects of the leaflet.** | | |
| We now come to the last question:  Please take a look at this early detection leaflet on SCS.  Tell us what you particularly like or dislike about this leaflet. | - Do you know the leaflet? - Do you have any suggestions for improvement? - Do you find it understandable? - Is all the information you need available or is something missing? If yes, what? - How do you find the layout on a scale of 1-10 (10 = very good)? - How clear/understandable/readable do you find it? | - Why? - Can you explain this further? - Can you give an example? - Can I ask again if I understood this correctly from you...? - Did I understand you correctly that...? - So your understanding is that... |
| - We have now reached the end of the interview. Is there anything else you would like to add or get rid of? | | |

- Distribution of the questionnaire
- Farewell
