# Supplementary material for: Increasing Participation Rates in Germany’s Skin Cancer Screening Program (HELIOS): Protocol for a Mixed Methods Study
Source: JMIR Res Protoc. 2021 Dec 13;10(12):e31860. doi: 10.2196/31860 (PMC8713106; doi:10.2196/31860)
Supplement: Multimedia Appendix 2 [file resprot_v10i12e31860_app2.docx]

**Haben Sie schon einmal von der freiwilligen Hautkrebs-Vorsorgeuntersuchung gehört ("Hautkrebsscreening")?**

🞏 Ja, durch (Mehrfachantworten möglich):

🞏 Arzt 🞏 Freunde/Bekannte

🞏 Krankenkasse 🞏 Zeitung

🞏 Fernsehen 🞏 Internet

🞏 Informationsbroschüre 🞏 Informationsplakat

🞏 Sonstige (Bitte um Angabe):

🞏 Nein

🞏 Weiß nicht

**Finden Sie die Möglichkeit zur Untersuchung von Leberflecken und Muttermalen gut?**

🞏 Ja 🞏 Nein 🞏 Weiß nicht

**Haben Sie schon einmal das Hautkrebsscreening in Anspruch genommen?**

🞏 Ja, bei (Mehrfachantworten möglich):

🞏 Hautarzt/Dermatologe

🞏 Hausarzt (Allgemeinarzt/Internist)

🞏 Betriebsarzt

🞏 Sonstiger Arzt (Bitte um Angabe):

zuletzt im Jahr

🞏 Nein, weil (Mehrfachantworten möglich):

🞏 ich kenne das Hautkrebsscreening bisher nicht

🞏 ich denke, das Hautkrebsscreening bringt nichts

🞏 ich hatte bisher keine Zeit

🞏 zu lange Wartezeiten für Termin

🞏 ich habe Angst vor Fehl-/Überdiagnosen

🞏 Sonstige Gründe:

🞏 Weiß nicht

**Hatte das Hautkrebsscreening einen Nutzen für Sie?**

🞏 Ja 🞏 Nein 🞏 Weiß nicht

🞏 Ich habe bisher noch nicht am Hautkrebsscreening teilgenommen

**Würden Sie das Hautkrebsscreening erneut in Anspruch nehmen?**

🞏 Ja

🞏 Nein, weil (Mehrfachantworten möglich):

🞏 Unzufrieden mit der Durchführung durch den Arzt

🞏 Fehldiagnose

🞏 ich bin generell nicht überzeugt, dass das Hautkrebsscreening sinnvoll ist

🞏 ich habe keine Zeit für solch einen Untersuchung

🞏 zu lange Wartezeit für einen Termin

🞏 sonstige Gründe:

🞏 Weiß nicht

🞏 Ich habe bisher noch nicht am Hautkrebsscreening teilgenommen

**Die gesetzlichen Krankenkassen in Deutschland erstatten seit 2008 allen Personen ab 35 Jahren alle zwei Jahre die Durchführung des Hautkrebsscreenings.**

**Würden Sie gerne alle zwei Jahre an die mögliche Inanspruchnahme des Hautkrebsscreenings erinnert werden?**

🞏 Ja, bevorzugt durch (Mehrfachantworten möglich):

🞏 E-Mail

🞏 Elektronische Kurznachricht, z.B. SMS

🞏 Anruf

🞏 Postalisch

🞏 persönliche Ansprache durch den Hausarzt oder Hautarzt

🞏 Kurzfilmnachricht

🞏 Krankenkasse

🞏 Bonusprogramm der Krankenkasse

🞏 Sonstiger Vorschlag:

🞏 Nein

🞏 Weiß nicht

**Würden Sie gerne alle 2 Jahre eine Einladung zum Hautkrebsscreening erhalten, in der Ihnen bereits ein Termin bei Ihrem Arzt vorgeschlagen wird?**

🞏 Ja 🞏 Nein 🞏 Weiß nicht

**Würden Sie gerne zusätzlich zu einer Erinnerung weitere Informationen zum Hautkrebsscreening (z.B. eine Informationsbroschüre) erhalten?**

🞏 Ja 🞏 Nein 🞏 Weiß nicht

**Glauben Sie, dass durch das Hautkrebsscreening Hautkrebs im Durchschnitt früher erkannt werden kann?**

🞏 Ja 🞏 Nein 🞏 Weiß nicht

**Wünschen Sie mehr Aufklärung durch Ihren Hautarzt/Hautarzt zum Hautkrebsscreening?**

🞏 Ja 🞏 Nein 🞏 Weiß nicht

**Angaben zu Ihrer Person:**

**Sind Sie männlich oder weiblich?**

🞏 Männlich 🞏 Weiblich

**Bitte geben Sie Ihr Alter an:**

Jahre

**Bitte geben Sie Ihren Familienstand an:**

🞏 ledig 🞏 geschieden

🞏 verheiratet zusammen lebend 🞏 verwitwet

🞏 verheiratet getrennt lebend

**Was ist Ihr höchster erreichter Bildungsabschluss?**

🞏 Kein Schulabschluss 🞏 Hauptschul-/Volksschulabschluss

🞏 Realschulabschluss 🞏 Sonderschulabschluss

🞏 (Fach-)Abitur 🞏 Hochschulabschluss

🞏 Anderer Schulabschluss:

**Bitte geben Sie Ihren derzeitigen Beruf an:**

**Bitte schätzen Sie wie lange Sie sich durchschnittlich in den Frühlings- und Sommermonaten (Anfang März bis Ende August) im Freien aufhalten:**

🞏 nie

🞏 <10 Stunden pro Woche

🞏 10-20 Stunden pro Woche

🞏 20-30 Stunden pro Woche

🞏 30-40 Stunden pro Woche

🞏 >40 Stunden pro Woche

**Hatten Sie bereits einmal Hautkrebs in Ihrer Krankengeschichte?**

🞏 Ja 🞏 Nein 🞏 Weiß nicht

**Wenn ja: Welche Art?**

🞏 Melanom („schwarzer Hautkrebs“) 🞏 Plattenepithelkarzinom (Spinaliom)

🞏 Basalzellkarzinom (Basaliom) 🞏 Andere:

**Hatte jemand aus Ihrer Familie bereits einmal Hautkrebs in der Krankengeschichte?**

🞏 Ja 🞏 Nein 🞏 Weiß nicht

**Hatte jemand aus Ihrem Bekanntenkreis bereits einmal Hautkrebs in der Krankengeschichte?**

🞏 Ja 🞏 Nein 🞏 Weiß nicht

**Schätzen Sie ihr eigenes Risiko an Hautkrebs zu erkranken als hoch ein?**

🞏 Ja 🞏 Nein 🞏 Weiß nicht

**Wie oft nutzen Sie ein Solarium?**

🞏 nie

🞏 im Durchschnitt einmal pro Woche

🞏 im Durchschnitt einmal pro Monat

🞏 im Durchschnitt einmal pro Woche

**Wie sind Sie derzeit krankenversichert?**

🞏 gesetzlich versichert

🞏 privat versichert

🞏 keine Versicherung

**Sie sind nun am Ende des Fragebogens angelangt.** **Vielen Dank für Ihre Teilnahme!**
